# Growth and Morphology of PbSe Mesocrystals

**DOI:** 10.1021/acs.cgd.5c00329

**Published:** 2025-05-21

**Authors:** Paolo Accordini, Joeri Takke, Willem J. P. van Enckevort, Elias Vlieg

**Affiliations:** Institute for Molecules and Materials, 6029 Radboud University, Heyendaalseweg 135, 6525 AJ, Nijmegen, The Netherlands

## Abstract

This work describes the growth, (surface) morphology, and defect structure of mesocrystals composed of lead selenide (PbSe) nanocrystals enveloped by a corona of oleate ligands. Three crystal growth methods are explored to reproducibly obtain mesocrystals up to tens of micrometers in size. The best results are obtained by vapor in-diffusion of a suited antisolvent into a PbSe nanocrystal suspension. This yields octahedral and trigon-shaped cubic close-packed (fcc) crystals composed of partially oriented nanocrystals. The morphology and surface structure of the mesocrystals are investigated by scanning electron microscopy and atomic force microscopy. This reveals a wealth of features, including various kinds of single and multiple {1 1 1} twinning, monolayer, and multilayer growth steps, the lower ones often being undulated by impurity blocking, ⟨1 2 1⟩ and ⟨1 1 0⟩ striations formed by a postgrowth phase transformation, linear growth faults, slip lines, and cracks. These observations show that the growth and defect structure of PbSe mesocrystals, for the most part, follow the classical crystal growth concepts, which are based on molecular or atomic growth units. The absence of spiral growth is a notable exception.

## Graphical abstract

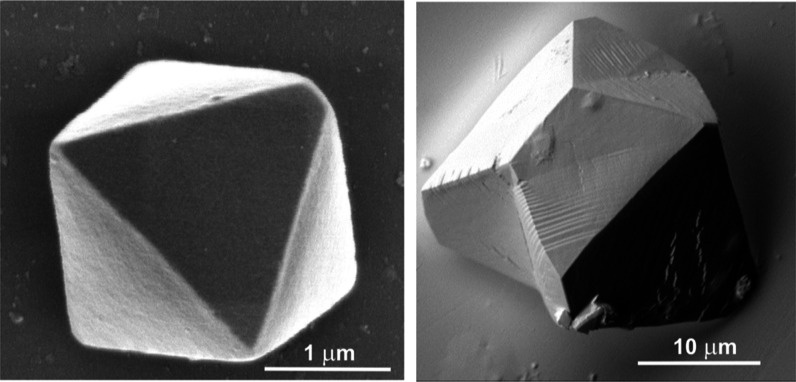

## 1 Introduction

Crystallization is an important process in nature. It can be understood to be composed of two major stages: first, the aggregation of elementary units to form a nucleus from a supersaturated solution and, second, its growth by the addition of elementary units to the nucleus. Ultimately, these are pathways that nature uses to minimize the overall free energy of the system. These processes span various orders of magnitude, from the atomic scale up to and beyond the micrometric scale. When classic crystallization pathways, involving atoms or small molecules, are compared against crystals composed of proteins or macromolecules, it is possible to observe differences in nucleation and growth because of the different ways in which elementary units can interact with each other and the differences in their environment.^1–3^

Much attention has been paid to the crystallization mechanisms of proteins and other biomacromolecules.^4^ The crystallization of these compounds shows many similarities with classical systems, such as two-dimensional nucleation and roughening. However, there are also differences, such as the rarity of spiral growth, which is very common in classical systems, and step generation starting from three-dimensional nuclei precipitated on the crystal surface.^4,5^ This latter, unique mechanism was observed for the growth of the (1 1 1) face of the satellite tobacco-modified virus crystal. Also, the rough growth by random deposition of growth units, as observed for lysosomes and catalase crystals, is rare for classical growth from solution.

Another class of systems studied in recent years is formed by mesocrystals composed of nanoparticles. Like in the case of biomacromolecules, the characteristics of this system are dictated by the shape, surface properties, and surface potential of the elementary unit. In this case, the particle is bigger than a single atom but (much) smaller than a protein or a small virus. The advantage, in this case, is that many of the particle parameters can be tweaked during synthesis, allowing for a more systematic exploration of the phenomena itself.

Since the early study by Bentzon et al.^6^ on ordered arrays of iron oxide nanoparticles imaged by electron microscopy, research on supercrystals composed of nanocrystals has soared. Excellent reviews on this issue are given in refs 7–10 . Ordered superstructures composed of nanometer- and submicrometer-sized crystals are ubiquitous, even in outer space.^11^ The formation of crystals by particle attachment plays an important role in geology and mineralogy, microstructures of biominerals in organisms, and the synthesis of a new class of materials.^7–10^ In our study, we examine to what extent these classical growth concepts can be applied to the specific case of mesocrystal growth. Following the definition by Niederberger and Cölfen,^7^ a mesocrystal consists of a self-assembly of nanocrystals embedded by a corona of (often) organic ligands from a dispersed state in solution. As a model compound to study mesocrystal growth, we used lead selenide (PbSe) nanocrystals.

Chalcogenide-based mesocrystals, composed of lead sulfide (PbS)^12–14^ or cadmium-selenide (CdSe)^15,16^ nanocrystals, have already been studied. In the PbS superstructures, the elementary building blocks are single cuboctahedral PbS nanocrystals embedded by oleate ligands with a diameter ranging from 3 to 10 nm.^12^ For CdSe, the basic units are embedded hexagonal close-packed CdSe nanocrystals. These studies were focused on growth methods,^16^ structure,^13,14^ the relation between nanocrystal orientation and superlattice array,^16,17^ morphology and twinning,^12^ and other defect formation. Methods used to characterize these mesocrystals are dominated by scanning electron microscopy (SEM), transmission electron microscopy (TEM), and X-ray diffraction.

In contrast to PbS and CdSe mesocrystals, PbSe-based crystals received much less attention. This compound was extensively used for detailed studies of 2D oriented attachment.^18–20^ Oriented attachment implies fusion of nanocrystals by direct bonding of specific nanocrystal facets without ligands at the interface and is thus different from mesocrystals. The lack of studies on this compound can partly be explained by the tendency of PbSe to oxidize under ambient conditions. Lead selenide quantum dots are more prone to oxidation effects when compared to PbS or CdSe quantum dots: this side effect ultimately triggers agglomeration and causes quantum dots to precipitate from the solution under ambient conditions. Given the time scale required for the growth of mesocrystals compared to that for oxidation of nanoparticles, a colloidal system will completely segregate from the solution before any mesocrystals are formed, unless the environment is free from oxygen.

One interesting study on this mesocrystal was focused on the occurrence and transitions of three different polymorphs and their thermodynamic properties.^17^ In our study, we investigate the formation of PbSe 3D mesocrystals, where the oleate ligands are not removed and where thus oriented attachment should not occur. We compare three different growth methods using different combinations of nanocrystal solvents and antisolvents. The morphology, surface, and defect structures of the crystals obtained are examined by SEM and atomic force microscopy (AFM). A main conclusion from our work is that the classical concepts of crystal growth and defect formation are well applicable to the nonclassical system of PbSe mesocrystal growth.

## 2 Experimental Methods

### 2.1 Growth

#### 2.1.1 Quantum Dot Synthesis

As a source material for the PbSe mesocrystal growth, lead selenide nanocrystals are synthesized using an adapted version of the method of Steckel et al.^21^ Details on materials used are given in the Supporting Information, see Supporting Information S1 and S2. Typical nanocrystal core diameters are 5.4 nm; including the oleic acid ligands, the diameter becomes 8.7 nm, as was estimated by TEM of 2D layers (Figure S6) and infrared spectroscopy.^22^ Three different methods are used to grow mesocrystals composed of PbSe: (i) solvent evaporation, (ii) liquid diffusion of the antisolvent, and (iii) vapor diffusion of the antisolvent (Figure 1a–c).

**Figure 1:**
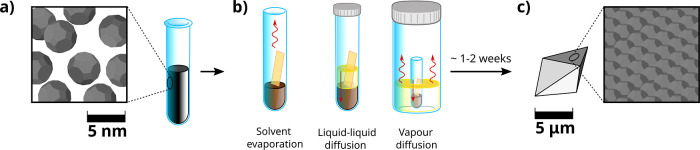
Schematic description of the mesocrystal’s synthesis—starting from the stock sample (a), experiments are prepared by diluting the solution (brown liquid) and by adding a mica substrate (orange plate in figure). Antisolvent was also added in a subset of the experiments (yellow liquid). Three different experiments were performed (b), from left to right: evaporation of the carrier solvent, diffusion of the liquid antisolvent, and diffusion of the antisolvent vapors. Note that only in the first case vials were not sealed. Straight red arrows highlight diffusion of the antisolvent, while the wiggly arrows denote evaporation. By waiting 1–2 weeks (c), NCs self-assemble into different structures deposited on the substrate, with an average size of several μm.

#### 2.1.2 Mesocrystal Growth Using Solvent Evaporation

Toluene and hexane solutions of 10^–7^ mol to 10^–5^ mol PbSe nanocrystals were prepared and stored inside a glovebox (<1 ppm of O_2_ and <1 ppm of H_2_O in a N_2_ atmosphere). Evaporation was carried out by simple evaporation of 1 mL solution in 2 mL test tubes kept at a constant temperature of 20 °C. Mesocrystals were grown on the surface of a small, tilted strip of muscovite mica placed at the bottom of the tube. The choice of this substrate was dictated by the combination of multiple factors: low cost, exceptionally flat substrate, and chemically inert. Evaporation was slow and took up to 4 weeks, after which the mica substrate was lifted from the container, cut in pieces, and examined by SEM.

#### 2.1.3 Mesocrystal Growth by Diffusion of the Liquid Antisolvent

For this method, a piece of mica was placed in a test tube. The solution with the largest density, thus either the antisolvent or the diluted nanocrystal solution, was placed in the test tube first and then the other one was added on top. 1 mL of diluted nanocrystal solution and 2 mL of antisolvent (Table 1) were used for all experiments. The vial was then sealed and carefully stored in a glovebox. The time necessary for complete mixing varied depending on the choice of the antisolvent and was usually between 2 and 5 days. The experiment was considered done when the originally dark nanocrystal solution was completely cleared out, and point-like aggregates were visible to the naked eye on the side wall of the vial. Then, the mica substrate was separated from the solution, dried, and cut in small strips for further examination using SEM. For several antisolvents, the solution did not clear, and no mesocrystals are formed.

**Table 1:** Results of PbSe Mesocrystal Growth by the Liquid–Liquid Diffusion Method and the Gas–Liquid Diffusion Method^a^

antisolvent	dipole moment	dielectric constant	mesocrystals from liquid–liquid diffusion	mesocrystals from vapor diffusion	
1,4-dioxane	0.1	2.3	N	Y	aprotic
chloroform	1.1	4.8	N	-	
diethyl ether	1.2	4.3	N	-	
*tert*-butyl methyl ether	1.3	2.6	N	-	
diisopropyl ether	1.5	3.8	N	-	
tetrahydrofuran	1.6	7.6	N	-	
ethyl acetate	1.8	6.0	Y	-	
oleic acid	2.0	2.5	N	-	
pyridine	2.2	12.4	Y	-	
acetone	2.9	20.7	Y	-	
dimethylformamide	3.8	36.7	Y	Y	
acetonitrile	3.9	37.5	Y	Y	
nitrobenzene	4.2	34.8	N	-	
					
ethanol	1.4	24.0	Y	Y	protic
1-propanol	1.6	20.0	Y	Y	
1-butanol	1.7	32.7	Y	Y	
methanol	1.7	32.7	Y	Y	
2-propanol	3.1	20.0	Y	Y	

a Each row represents an experiment prepared as described in the Experimental Methods section using hexane as a solvent and the listed compound as an antisolvent. Items marked with - were not tested with that specific pair of method/antisolvent.

#### 2.1.4 Mesocrystal Growth Using Diffusion of Antisolvent Vapors

In a 20 mL vial, 2 mL of antisolvent was placed. 1 mL solutions of nanocrystals of known concentrations were prepared in a small, 1.5 mL test tube. A strip of mica was added, and then the assembly was placed in the larger tube containing the antisolvent. The inner vial was left open, and the bigger one was sealed. The system (composed of the bigger vial with the smaller one inside) was kept in the glovebox until the solution in the smaller vial was cleared. This took usually 1–2 weeks. After that, the mica was separated from the solution and cut into pieces for further study. Like the case of diffusion of liquid antisolvent growth, several antisolvents did not clear the solution, and no mesocrystals were formed.

Toluene and hexane, in combination with a range of different antisolvents, were used as a solvent in both diffusion of liquid antisolvent and diffusion of antisolvent vapor approaches.

### 2.2 Characterization

#### 2.2.1 Morphology, Surface, and Bulk Structures of the PbSe Mesocrystals

The mesocrystals were characterized using SEM, AFM, and X-ray diffraction. The crystals had to be handled with care, as the specimens deform easily under application of stress, as shown in Supporting Information S3.

X-ray diffraction was done on a single PbSe mesocrystal using a Bruker D8 Quest diffractometer employing Cu Kα_1_ radiation (Supporting Information S4).

Scanning electron microscopy was performed by using different SEM apparatuses. For medium magnifications, a Phenom-World microscope was used. Higher magnifications were obtained by using a Jeol 6330 FESEM or a Zeiss sigma 200 FESEM. Prior to examination, the crystals were coated by an ultrathin metal layer using a sputter coater, which did not affect the crystal surfaces.

AFM measurements were carried out using a Digital Instruments Dimension 3100 AFM, utilizing NGS-10 tips with a tip radius of 10 nm. Most measurements were performed in the intermittent contact (“tapping”) mode.

## 3 Results and Discussion: Growth

### 3.1 Evaporation

Evaporation-induced self-assembly gave unreliable results, for both hexane and toluene as a solvent. When evaporating the solvent, the nanocrystals did not grow into ordered mesostructures, but formed planar, often branched aggregates (Figure 2, left). The SEM images showed no faceted crystals but layers of many micrometers thickness with “holes” on the substrates. In one case, starting from a 0.525 μmol solution in toluene, nanocrystals managed to assemble in faceted, octahedral mesocrystals (Figure 2, right). However, repeating the experiment under comparable conditions yielded different outcomes. The evaporation method was abandoned in the rest of this study due to these complications. For many crystals, slow growth conditions give the best results, but in this case, where growth takes several weeks, this is not true. The reason is most likely the high solubility of the nanocrystals, leading to a too high concentration at the end of the experiment when nearly all solvent has evaporated and growth takes place.

**Figure 2:**
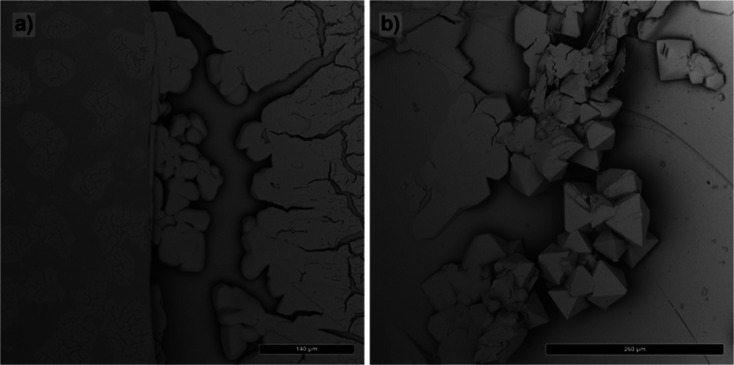
Morphologies of crystals grown by the evaporation method. (a): nonfaceted planar aggregates and (b): faceted octahedrons.

### 3.2 Antisolvent Diffusion

#### 3.2.1 Influence of Antisolvent

Mesocrystals can also be grown by diffusion of an antisolvent liquid into the nanocrystal solution, either by liquid to liquid or by vapor to liquid transport. In parallel with normal crystal growth,^23^ the solubility of nanocrystals can be lowered by adding a suited antisolvent, miscible with the solvent in use. Roughly speaking, this is a consequence of an increase in the nanocrystal–solution interfacial energy upon addition of the antisolvent. This favors the formation of solid–solid bonds between adjacent nanocrystals, overcoming their entropic dispersion in the solution and thus promoting mesocrystal growth. Using diffusion of the liquid antisolvent has proven to be successful in the growth of PbS^13^ and CdSe^16^ mesocrystals. Compared to the evaporation method, diffusion of the liquid antisolvent and diffusion of the antisolvent vapor result in far better PbSe mesocrystals, with isolated and well-faceted assemblies.

The basic shapes of the PbSe mesocrystals are octahedra and trigons, as shown in Figure 3. In addition, different kinds of twins are formed. Details on morphology, twinning, and defect formation will be given in Section 4. Apart from size, discussed below, there were no essential differences in crystal shape for the two antisolvent diffusion methods used.

**Figure 3:**
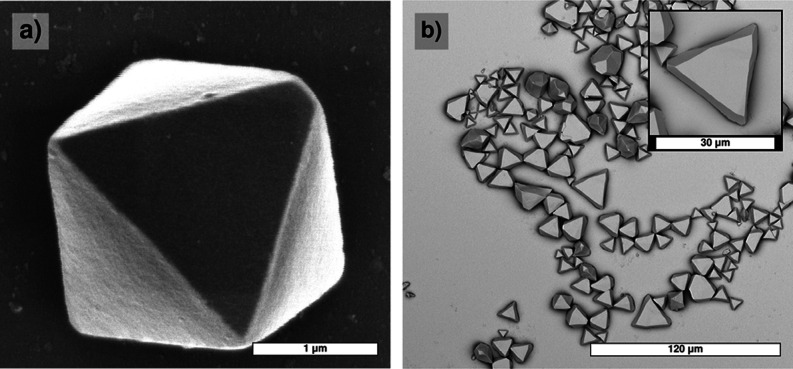
Two basic shapes of the PbSe mesocrystals obtained by antisolvent diffusion. Both shapes consist of {1 1 1} faces, where the trigon can be considered a slice cut below one octahedron face. (a): octahedrons; (b): trigons.

The main constraints on suitable solvent/antisolvent pairs are miscibility and polarity. Both substances must have some degree of miscibility in order to allow diffusion of the antisolvent from the liquid or vapor phase into the solution. In addition, for the vapor diffusion method, the vapor pressure of the antisolvent liquid must be sufficiently high (around 1 kPa or higher). As solvents, we used hexane and toluene, which gave similar results. Runs using these solvents and different antisolvents, both aprotic and protic, were performed employing both the diffusion of the liquid antisolvent and the diffusion of antisolvent vapor methods. Results for hexane solutions, including the dipole moments and dielectric constants of the antisolvents, are given in Table 1.

From Table 1, it is clear that the antisolvents with higher dipole moments, D, favor mesocrystal formation. The nanocrystals are covered by Pb-oleate ligands, with the aliphatic part pointing outward. The outer end points of these ligands resemble long chain aliphates, which typically have a dipole moment of 1.7 D. If the dipole moment of the antisolvent is larger than this value (Table 1), mesocrystals are formed. For the antisolvents of low dielectric constant, *D* ≤ 1.7, the nanocrystals remain dissolved, no mesocrystals are formed, and the solution is not cleared. For pure hexane (1.7 D) and toluene (1.0 D), this difference is minimal: nanocrystals dissolve very well, and mesocrystals are formed only after almost complete solvent evaporation, when the concentration is too high to form well-ordered structures.

For increasing dielectric constant of the antisolvent, which is attributed to a higher dipole moment or to the presence of hydrogen bonds, the nanocrystal solubility decreases and mesocrystals are formed. For both the aprotic and protic antisolvent molecules, this is a consequence of the hydrophobic effect.^24–27^ Here, strongly polar molecules or molecules forming hydrogen bonds rearrange when they come into contact with an apolar surface. This leads to a local increase in ordering of the solvent molecules adjacent to the surface, which lowers the entropy and thus increases the interfacial Gibbs free energy. This increase in the surface free energy is reinforced by a partial rupture of the strong dipole–dipole interactions or hydrogen bonds of the solvent molecules in contact with the apolar surface, which is an extra, enthalpic, contribution. As the surface of the PbSe nanocrystals is enveloped by a shell of oleate ligands with their apolar tails pointing outward, this surface can be considered as apolar, and the hydrophobic effect will happen here as well. For the aprotic molecules, this effect results from the antisolvent dipole–dipole interactions; for the protic alcohols, this effect is taken over by hydrogen bond formation, and the low dipole moments do not play a role. The increase in the interfacial free energy of the isolated nanocrystals in solution leads to their accumulation into mesocrystals, lowering the total surface free energy.

The hydroxide group of the alcohols may play an additional role by replacing the oleate ligands, leading to direct attachment of the nanocrystals, as was found for the 2D systems in the presence of ethylene glycol.^18–20^ However, this does not play an important role in our experiments since well-formed mesocrystals with unaffected surfaces are obtained using alcohols as an antisolvent. The exceptional behavior of nitrobenzene is possibly explained by oxidation effects as this molecule is a mild oxidizer.^28^

Notably, 1,4-dioxane is an exception to the observed trends, and we did not find an explanation for this particular case.

#### 3.2.2 Average Size

In contrast to the general morphologic features, the size of the mesocrystals does vary with the growth method, the antisolvent used, and the initial nanocrystal concentration in the solution.

Unlike the protic antisolvents, for the aprotic antisolvents, the mesocrystal size increases with initial nanocrystal concentration. As expected, both for the protic and aprotic antisolvents, crystal growth proceeds slower by using the diffusion of the antisolvent vapor method as compared to diffusion of the liquid antisolvent because the vapor transport is slower than advection at the liquid interface. This was concluded from the longer solution clearing times in the first case. Acetonitrile produces the largest crystals; therefore, this aprotic antisolvent was used to measure mesocrystal size as a function of the initial nanocrystal concentration in toluene. Results are displayed in Figure 4 for both the diffusion of liquid antisolvent and the diffusion of antisolvent vapor cases.

**Figure 4:**
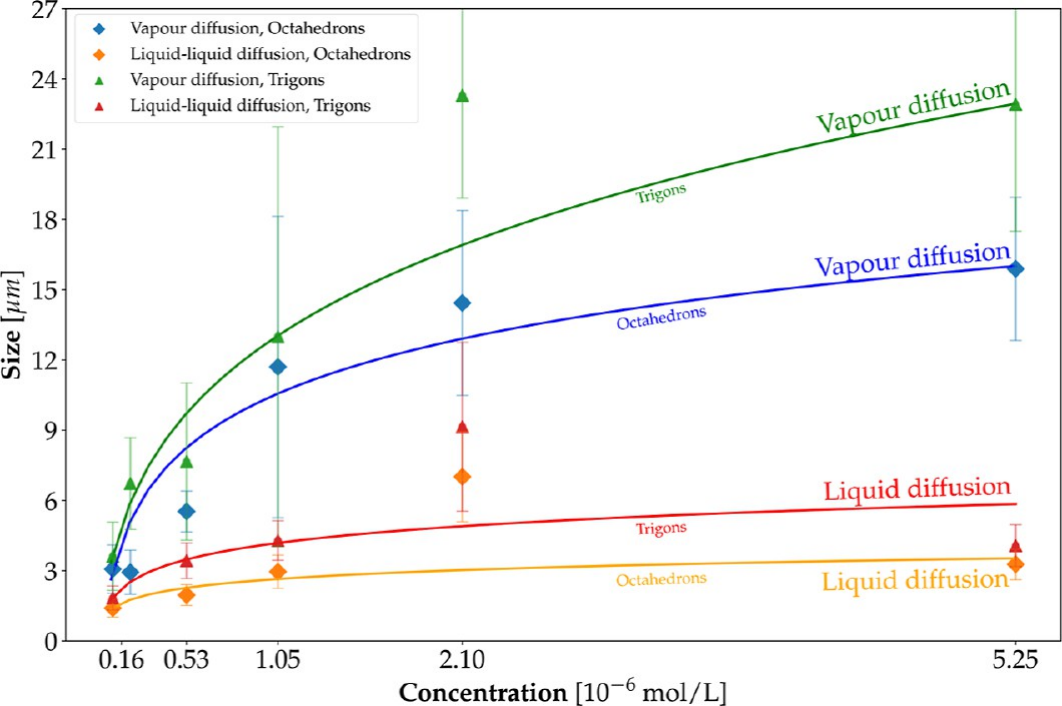
Average size of PbSe mesocrystals as a function of initial nanocrystal concentration in toluene grown by diffusion of liquid antisolvent (blue dots) and diffusion of antisolvent (orange dots) vapors. Graphs are shown for the two most common species, trigons and octahedrons, found in the sample. The simple model given in the text (blue dotted line for diffusion of liquid antisolvent and orange dotted line for diffusion of antisolvent vapors) works reasonably well. In both cases, diffusion of antisolvent vapors produced larger structures.

In these figures, the initial nanocrystal concentrations in the toluene solutions are expressed in micromoles and are determined by infrared spectroscopy.^22^ Octahedrons as well as trigons were measured. For the octahedrons, the size is determined by the edge length, and for the trigons, it is the length of the outer edge of the triangular crystals (see also Supporting Information S5). The average sizes and the associated standard deviations are obtained by more than 100 size measurements of the crystallites on the mica substrates obtained after each run. The ratio of the outer and inner trigon edge lengths is 1.3 for all cases (Figures 3a and S5).

From the results shown in Figure 4, it is clear that the crystal sizes are considerably larger for the diffusion of antisolvent vapor experiments as compared to the diffusion of liquid antisolvent runs. In both cases, we see a reduction in the increase of crystal size for larger nanocrystal initial concentrations. The size, *s*, versus initial concentration, *c*, roughly follows

$$s=kc^{1/3}$$

for the diffusion of antisolvent vapor system as drawn in Figure 4. *k* is a constant, which is ≈11 μm/μmol^1/3^ for the octahedrons and 15 μm/μmol^1/3^ for the trigons. As the length of the inner trigon edges is 1.3^–1^ times the outer edge lengths, the size of the trigon top faces is similar to that of the octahedron faces, i.e., *k* ≈ 15/1.3 = 11.5 ± 1 μm/μmol^1/3^. This implies that the growth rates of the trigon top faces are close to that of the octahedron faces. As will be discussed in Section 4, the trigons are nucleated on the mica substrate, while the majority of the octahedrons is formed in the solution.

The dependence of crystal size on *c* ^1/3^ points to a brief, early nucleation period generating N nuclei followed by crystal growth. Here, N is assumed to be roughly independent of the initial nanocrystal concentration. This sudden nucleation takes place when sufficient antisolvent has entered the nanocrystal solution. At the end of the experiment, the solution is cleared, and virtually, all solute has been consumed by subsequent growth of the initial nuclei. Therefore, *Ns* ^3^ ∝ *c* and thus *s* ∝ *c* ^1/3^.

Moreover, since the nucleation is caused by an early destabilization of the colloidal solution, this also explains the difference in average sizes found between the two methods. The rate of diffusion of antisolvent using the vapor method is much smaller than the direct mixing and diffusion employed in the liquid–liquid one. Such a rapid change in solubility triggers the formation of more nuclei, compared to a diffusion from vapors. More nuclei mean that there are fewer nanocrystals available per mesocrystal, leading to a smaller size.

## 4 Results and Discussion: Morphology

### 4.1 Crystal Shape

Clean crystal shapes and surfaces suitable for investigation by SEM were obtained by both the diffusion of liquid antisolvent and diffusion of antisolvent vapor methods. For AFM, mainly crystals grown by diffusion of antisolvent vapor were used. Prior to microscopic examination, the crystals were harvested after the solution became clear. Because of the antisolvent, the concentration will be very low: this largely reduces the shut off effect,^29^ i.e., the uncontrolled growth of the specimens during separation from the solution. No essential differences in the morphology were found among the different antisolvents used.

The mesocrystals are always bounded by {1 1 1} faces, and in a few cases, also small {1 0 0} faces were observed. This leads to octahedrons and trigons, which are not twinned, 2-fold twins, mackles, 5-fold twins, and some icosahedral twins (Figures 3 and 5a). Similar single and multiple twinning have been encountered and studied by Rupich et al.^12^ for PbS mesocrystals. The twin plane is {1 1 1} in all cases. The growth is nonepitaxial with respect to the mica substrate. Nucleation of the crystallites preferentially occurs along the higher mica cleavage steps (Figure 5f, last picture). The trigons elaborated in the previous section show an exact {1 1 1} orientation parallel to the substrate, and these crystals are likely nucleated directly onto the substrate (Figures 3a and 5a). In many other cases, often truncated, octahedral crystals are formed by precipitation of nuclei from the mother solution onto the substrate. This was concluded from the length ratio of the ⟨1 1 0⟩ ridges between the (1 1 1) top face and an adjacent {1 1 1} side face and the contact line of this second plane with the substrate being larger than 2.^30^ For the trigons, this ratio is 0.67, confirming direct nucleation on the substrate.

**Figure 5:**
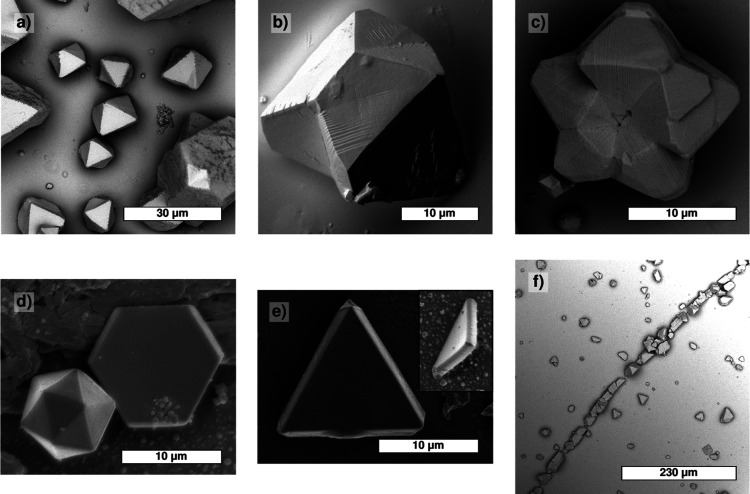
Different morphologies of PbSe mesocrystals deposited on (0 0 1) muscovite mica (SEM). (a) Nontwinned octahedrons; (b) singly twinned crystal; (c) 5-fold twinned crystal; (d) thin, twinned, hexagonal crystal (mackle) and icosahedron; (e) twinned, flat triangular crystals; and (f) heterogeneous nucleation of crystallites along a cleavage step on mica. Mesocrystals shown in (b,c) exhibit striated patterns parallel to [21̅1̅ ] on (1 1 1).

The morphology bounded by {1 1 1} faces is typical for an fcc lattice. In the literature, also bcc lattices have been reported,^17^ but these are expected to show a different morphology bounded by {1 1 0} faces^31^ and twinning along {1 1 2}.^32^ A dodecahedral morphology bounded by {1 1 0} has been observed for bcc PbS crystals by Huang et al.^33^ X-ray diffraction (Figure S4) shows that the nanocrystals in the mesocrystal are only partially orientationally ordered.

### 4.2 Striations

A considerable amount of the crystals show striated patterns that traverse the whole crystal (Figures 5b,c and 6a,b). At the front {1 1 1} surface of the octahedrally shaped crystals (indicated by f in Figure 6c), these patterns run parallel to a ⟨1̅ 2 1̅⟩ direction. For the {1 1 1} side faces (s in Figure 6), these lines are parallel to the ⟨1 1 0⟩ direction. These features are the outcrops of an array of planar faults parallel to a given {1 1 0} plane intersecting the different {1 1 1} faces of the octahedrons. Closer examination shows a zigzag sloping surface of which the alternating slopes are bounded by the parallel outcrops of these faults. The relative slope with respect to {1 1 1} is about 10°. No interaction with growth steps on the surface was observed, so the zigzag fault patterns must have been formed after growth. For several crystals, different domains of striated patterns are formed. On the same {1 1 1} surface, these different domains of striations are oriented 60° with respect to each other and do not overlap (Figure 6a). All these observations point to a phase transition from the fcc structure toward two oppositely twinned phases bounded by a mirror plane parallel to a given {1 1 0} plane in the crystal. Such striations as a consequence of an internal structural transformation have, for example, also been observed for C70 crystals.^34^

**Figure 6:**
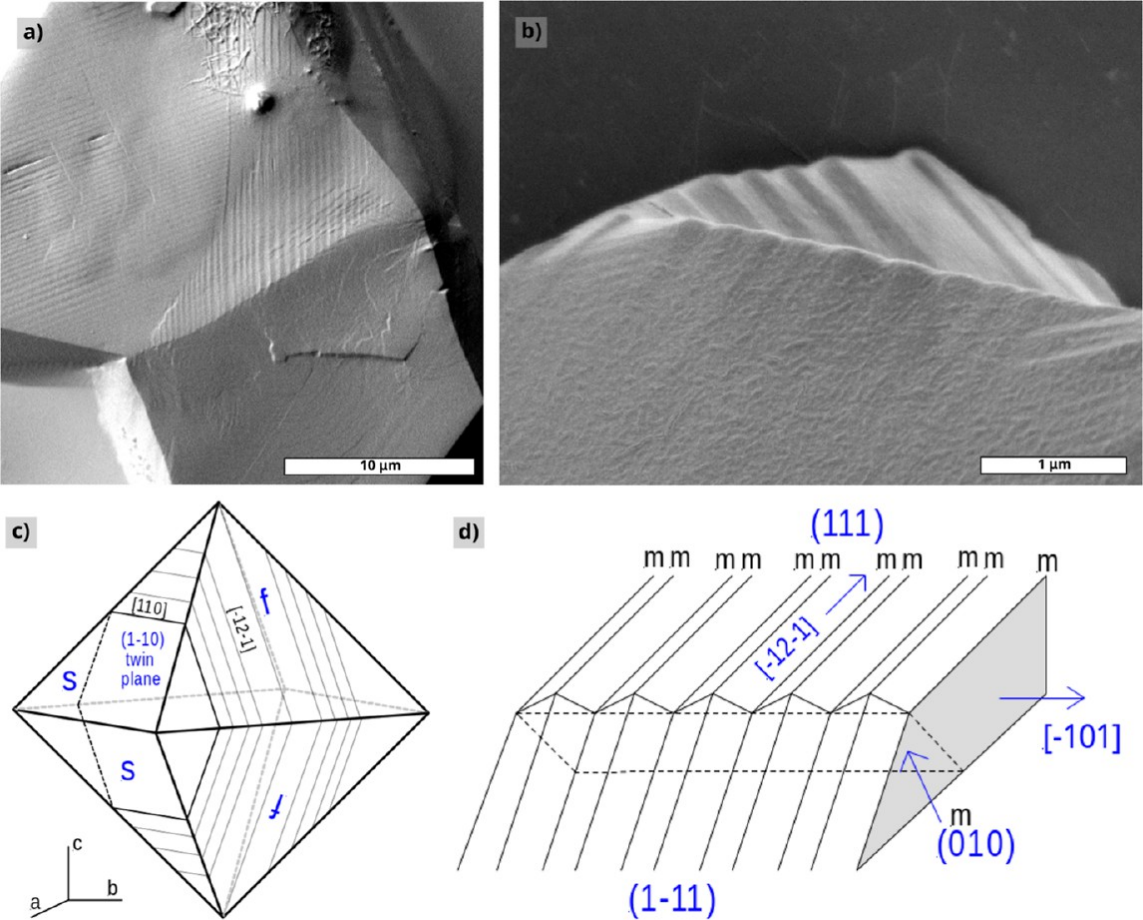
Zigzag striated patterns on the {1 1 1} faces of PbSe mesocrystals formed after growth (SEM). (a) Overview; (b) high-magnification view of the striation pattern crossing the front and side {1 1 1} face; (c) schematic view showing the outcrops of twin planes parallel to(11̅0) on the surfaces of a mesocrystal octahedron; and (d) schematic view of the zigzag pattern crossing two front {1 1 1} faces.

Several different phases have been reported for PbS and PbSe mesocrystals, being face-centered cubic (fcc), body-centered cubic (bcc), body-centered tetragonal (bct), and hexagonal closed-packed (hcp).^33,35,36^ These phases were identified by using synchrotron small-angle X-ray diffraction. By using solution calorimetry, Quan et al. found that for PbSe, the bcc phase is thermodynamically the most stable one, although the difference in (free) enthalpy of the various polymorphs is very small, with the value of 0.55 $\frac{kJ}{mol}$ or less.^35^

From this, it is expected that conversion between the different phases is easy. It is well known that conversion of an fcc to a bcc structure takes place via the Bain transformation path, with bct as an intermediate phase.^37,38^ This conversion proceeds by a simultaneous compression of the *c*-axis and an expansion of the a, *b* axes perpendicular to *c*, from *c*/*a*|_fcc_ = 1 to $c/a{|}_{fcc}=\frac{1}{\sqrt{2}}$ (or in bcc coordinates: $c/a{|}_{fcc}=\sqrt{2}$ to *c*/*a*|_fcc_ = 1) (Figure 7).

**Figure 7:**
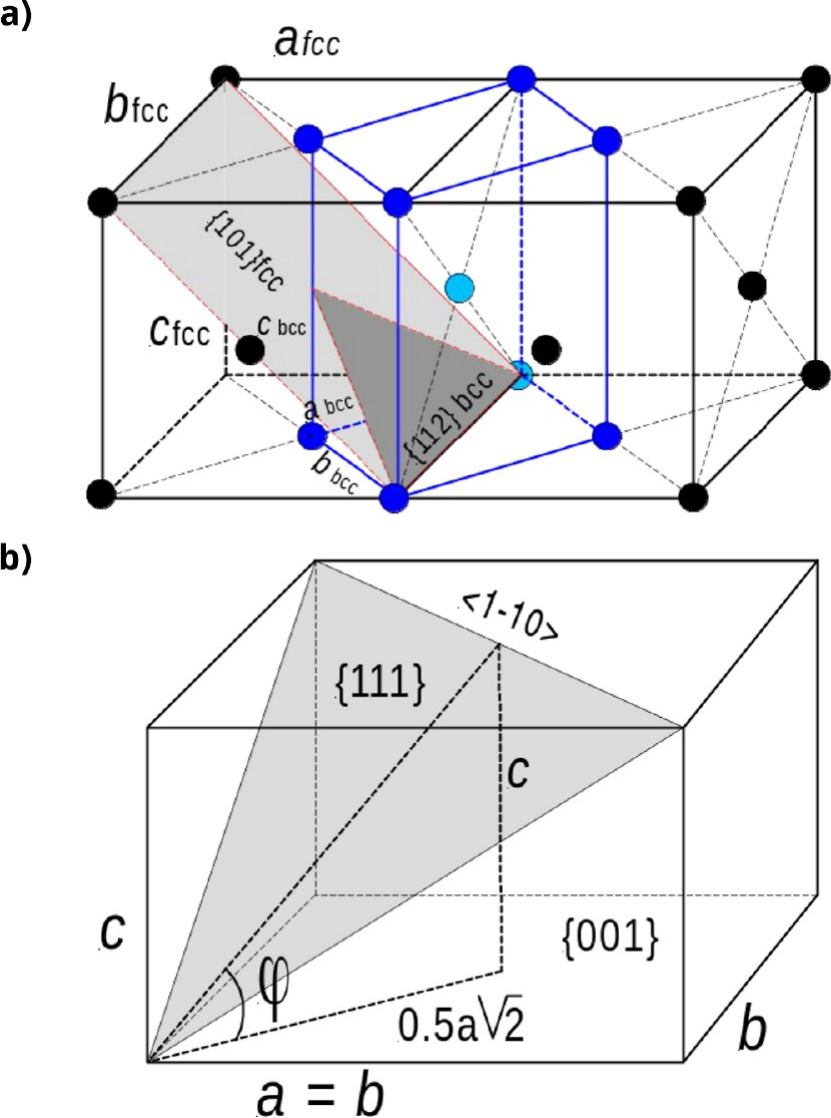
Bain transition from the face-centered cubic to the body-centered cubic crystal structure. (a) Structural diagram of the fcc and bcc structure; by compressing the *c*-axis and expanding the *a*, *b* axes, the fcc cell (black) turns into a bcc cell (blue). The twin plane {1 1 2} in bcc corresponds with the {1 1 0} plane in the fcc setting. (b) The orientation of the {1 1 1} plane as a function of $\frac{c}{a}$ ratio: $\varphi =\mathrm{atan}\left(\frac{c}{a}\sqrt{2}\right)$ .

The orientation of the {1 1 1} plane with respect to the basal {0 0 1} plane in fcc coordinates is given by $\varphi =\arctan\frac{a}{c}\sqrt{2}$ , as shown in Figure 7b. For fcc with *c*/*a* = 1, φ = 54.7°, for bcc with $c/a=\frac{1}{\sqrt{2}}$ , φ = 45°. This gives an orientational change of {1 1 1} of 9.7° during fcc to bcc transformation, which is close to the observed value of about 10°, the slope of the zigzag patterns. The observed {1 0 1} twins between the striation lamella are not expected for an fcc structure, which favors {1 1 1} twinning. However, it is well known that the most common twin plane in bcc crystals is {1 1 2} in bcc coordinates.^32,37^ This corresponds to {1 0 1} in a fcc setting as shown in Figure 7a. Assuming a postgrowth fcc to bcc transformation, this explains the observed {1 0 1} twin planes bounding the zigzag striation patterns observed in our PbSe mesocrystals. So, the striated crystals are bcc type, the most stable polymorph, which was formed after cessation of growth. This post growth transformation from fcc to bcc might be due to strain induced during handling of the crystals, slight heating in the SEM or evaporation of solvent after growth. In fact, we here have bcc structure crystals in a morphological fcc “jacket”.

### 4.3 Surface Morphology

#### 4.3.1 Steps

Growth steps, ranging in height from one lattice spacing, *d*_111_, to macrosteps and even minifacets^39^ are observed on the {1 1 1} faces by SEM and AFM (Figures 8 and 9). These observations indicate that the mesocrystals grow via a classical layer-by-layer mechanism. The step height ranges from (6 ± 3) nm (unit height steps, Figures 8a and 9a) to several hundreds of nanometers. The theoretical height, d_111_, of the unit steps is $d\frac{\sqrt{2}}{\sqrt{3}}$ , with *d* = 8.7 nm the nanocrystal diameter, including the ligands. This gives a height of ≈7.1 nm, which agrees with the observed value. The lower steps on the {111} faces are undulated and show no preferred orientation (Figures 8 and 9); the macrosteps and minifacets are oriented parallel to the ⟨1 1 0⟩ directions, of which the minifacets approach {1 1 1} planes (Figure 8b). The step spacing of the mono height steps ranges from 0.2 to several micrometers. No individual nanocrystals were observed either by SEM and AFM. This is likely due to the shut off effect at the end of the experiment^29^ and/or subsequent oxidation by the ambient, leading to a nanorough surface of ±1 nm in height as measured by AFM (Figure 9b). To demonstrate that AFM is capable of directly imaging separate nanocrystals, a drop-cast experiment was performed, which gave a positive result as shown in Figure S6.

**Figure 8:**
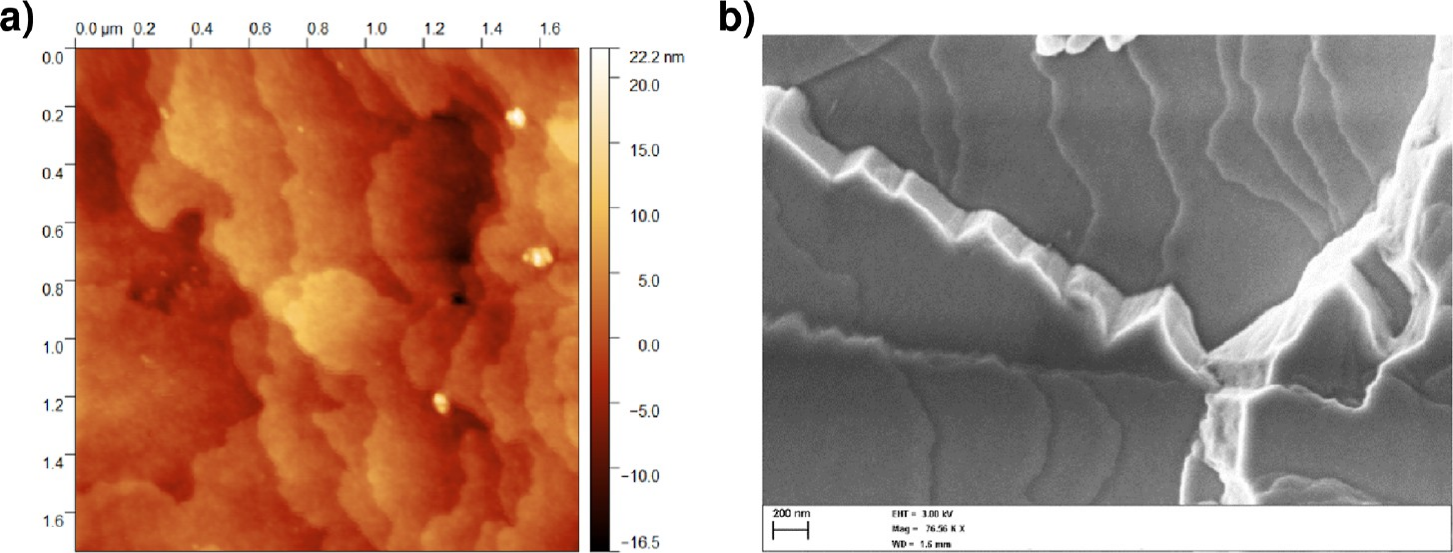
Steps on the {1 1 1} faces of PbSe mesocrystals. (a) Unit height steps (AFM) and (b) higher steps and minifacets (SEM).

**Figure 9:**
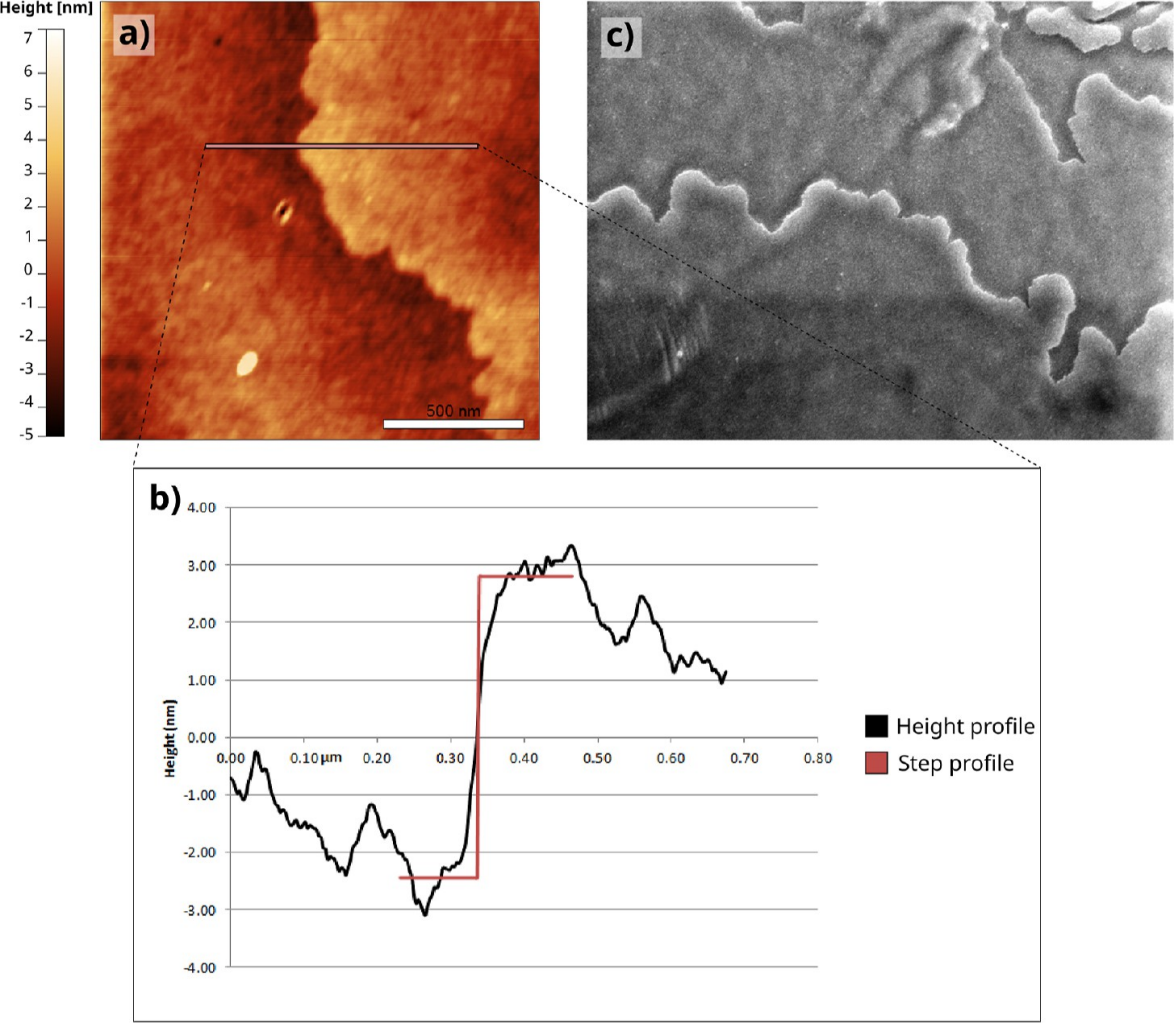
Capriciously shaped steps on the {1 1 1} faces of the PbSe mesocrystals. (a,b) Unit steps of height d_111_ (about 5 nm) imaged and measured by AFM and (c) somewhat higher steps recorded by SEM.

The lower steps show a capricious pattern, which is likely due to local blocking of their propagation by impurity pinning points in a similar way as observed for, among others, potassium dichromate crystals.^40^ This holds for both the lowest *d*_111_ and somewhat higher steps (Figures 8 and 9c). Local pinning of growth steps often leads to a supersaturation zone of no or retarded growth, especially at low supersaturations.^41–43^ In our case, the supersaturation was probably low at the end of the experiment, prior to removal of the crystals from the solution. This follows from the complete transparency of the solution, which is almost devoid of nanocrystals. The average separation of the pinning points is about 200 nm. Individual blockers could not be discerned on the AFM and SEM micrographs, so their identity remains unknown. The observed development of macrosteps is characteristic for this hampering of step growth by impurities as was shown by theory and simulation^44–46^ as well as by experiment.^39,47–49^ This supports our conclusion.

#### 4.3.2 Growth Mechanisms

No evidence is found for spiral growth^2^ neither by SEM nor by AFM. This is likely due to the very high dislocation burgers vector, *b*, of the PbSe mesocrystals. As the dislocation energy is proportional to *b* ^2^, its formation energy is high, and dislocations are not easily formed in this crystal with large lattice dimensions.^32^ Mesocrystals are grown at low supersaturation in order to obtain crystals of high quality. For normal crystals, the dominant growth mechanism is then spiral growth originating from screw dislocations. Since the mesocrystals do not show such defects, different growth mechanisms must be active. Sources of the steps on the {1 1 1} mesocrystal faces are twins, contact nucleation, and, likely, two-dimensional nucleation starting from the crystal edges. The very thin, triangular, and hexagonal mackle crystals (Figure 5d,e) are grown by the twin plane reentrant edge (TPRE) mechanism, introduced by the presence of one or more twin planes parallel to {1 1 1}.^50–53^ In this process, 2D nuclei, initiating step growth, are preferentially formed at the acute angle between the crystal faces adjacent to a twin outcrop, promoting their growth. For fcc crystals, a single twin is known to generate a triangular plate with its top and bottom face parallel to {1 1 1}.^50–52^ This was also found for our PbSe mesocrystals, as shown in Figure 5e. The three fast growing $\left\langle 2\overset{¯}{1}\overset{¯}{1}\right\rangle$ directions with a reentrant corner at the side faces have grown out, leaving the slow $\left\langle \overset{¯}{2}11\right\rangle$ trigon directions with an obtuse surface profile (inset Figure 5e). It is to be noted that these twinned trigonal crystal plates are different from the nontwinned trigons set out before. On the other hand, double, parallel twins lead to hexagonal plates (Figure 5d).^50–52^ Figure 10a shows a double twin on a hexagonally shaped crystal acting as a TPRE step source, from which steps propagate in opposite directions. Twin lines were also observed on the side faces of the ultrathin hexagonal crystals.

**Figure 10:**
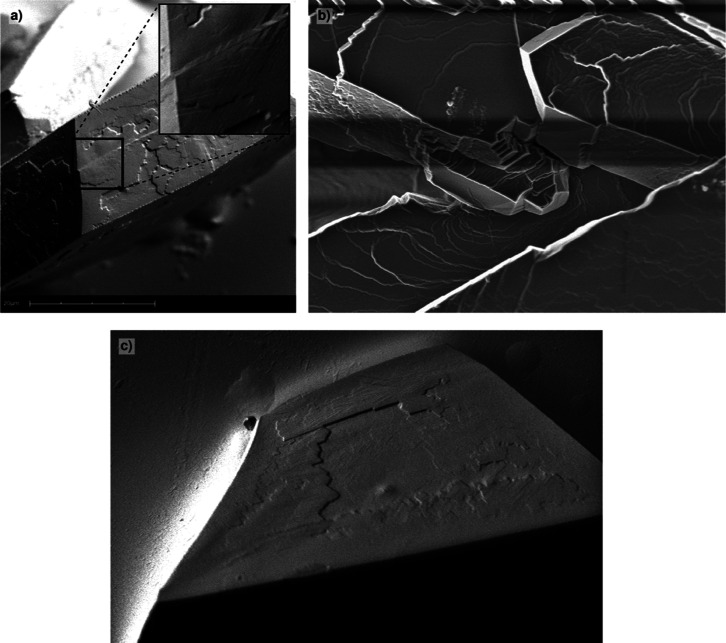
Different sources of steps. (a) Step generation from a double {1 1 1} twin by the TPRE mechanism (inset: pair of parallel twin outcrops in detail); (b) contact nucleation from foreign crystallites; and (c) generation of steps by 2D nucleation at the crystal edges.

A second, commonly observed, source of steps is contact nucleation^54^ induced by small crystallites deposited on top of the crystal surface (Figure 10b). A third source is 2D nucleation and subsequent growth starting from the crystal edges (Figure 10c). This is due to enhanced diffusion transport of the growth units that favors the edges of the crystals, leading to a locally increased supersaturation.^55,56^ Here, it should be realized that the diffusion coefficient of the nanocrystals is quite small, as it is inversely proportional to the particle diameter.^57^ For the highest initial supersaturations and largest crystals, this leads to a central hole at the surface of the crystal; see Figure S7.

#### 4.3.3 Linear Faults and Postgrowth Damage

Mesocrystals are very fragile, so their manipulation during analysis can easily lead to defects. For the largest crystals, faults outcropping at the {1 1 1} surfaces can be seen (Figure 11). The linear outcrops of these defects run parallel with either the $\left\langle \overset{¯}{1}01\right\rangle$ or the $\left\langle 1\overset{¯}{2}1\right\rangle$ directions on {1 1 1}. The $\overset{¯}{1}01$ faults are likely the outcrops of {1 1 1} slip lines^32^ induced by dislocation glide initiated by post growth shear stress (indicated by S in Figure 11a). This follows from the fact that these slip steps are perfectly straight and do not disturb the original growth step patterns. The situation for the $\left\langle 1\overset{¯}{2}1\right\rangle$ faults is less clear. They have a finite width and are bounded by two parallel lines (Figure 11). It is not a simple {1 1 1} slip or twinning, as in these cases, the line patterns should be parallel to $\langle \overset{¯}{1}01\rangle$ . Most of the $\left\langle \overset{¯}{1}21\right\rangle$ lines seem not to disturb the growth step patterns (Figure 11b), but exceptions are found (Figure 11c). The identity of the planar faults and the narrow domains associated with these defects is not clear. In case of twinning, the hypothetical twin plane ending on (1 1 1) should satisfy the zonal equation – *h* + 2*k* – *l* = 0.^58^ However, such a twin plane in the $\left[\overset{¯}{1}2\overset{¯}{1}\right]$ zone has, as far as known to us, not been reported for an fcc lattice.

**Figure 11:**
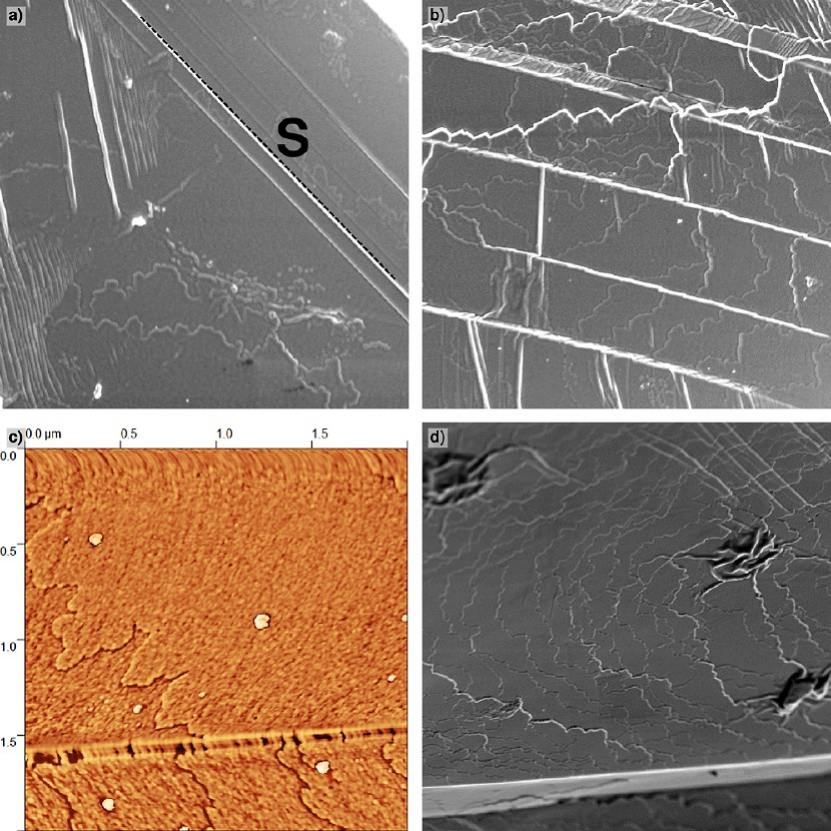
Linear faults and post growth damage (a,b,d: SEM; c: AFM). (a) Slip lines parallel to $\left\langle 1\overset{¯}{1}0\right\rangle$ induced by dislocation glide (S) and $\left\langle \overset{¯}{2}11\right\rangle$ faults; (b) narrow and wider $\left\langle \overset{¯}{2}11\right\rangle$ faults; (c) $\left\langle \overset{¯}{2}11\right\rangle$ fault in detail (AFM); and (d) post growth damage.

A number of crystals show cracks (Figure 11d) or linear fissions at the surface. This is likely due to the evaporation of enclosed toluene/hexane after separation from the growth system or in the vacuum during sputtering and SEM. It should be realized that the spaces between the large PbSe nanocrystals can host hundreds of solvent molecules. The free space per nanocrystal in the mesocrystal fcc lattice is

$$\frac{1}{4}V_{cel}-V_{nanocrystals}=\frac{1}{4}{\frac{4}{\sqrt{2}}}^{3}r^{3}-\frac{4}{3}\pi r^{3}=121{nm}^{3}$$

using a nanocrystal radius of 4.35 nm. The volume of one toluene molecule is estimated by

$$V_{tol}=\frac{M}{\rho_{tol}N_{av}}=0.177{nm}^{3}$$

with *M* the toluene molecular weight and ρ_tol_ the density of liquid toluene. This implies that up to 684 toluene molecules per nanocrystal can be stored in the lattice. In reality, the amount is likely less due to the hydrophobic effect of the antisolvent but is still quite substantial. (Partial) evaporation of these molecules, which also affects the ligand–ligand interactions between neighboring nanocrystals in the mesocrystal,^12^ leads to stresses in the crystals resulting in cracks, fissions, and glide. A similar phenomenon is well-known for protein crystals, where water evaporation after their separation from the aqueous solution leads to severe degradation of the specimens.^59,60^

## 5 Conclusions

Mesocrystals composed of PbSe nanocrystals embedded by oleate ligands are grown from solution on mica substrates to gather insight in the crystallization process of this nonclassical system. Aside from growth, attention is also paid to the bulk and surface morphology and defect formation. Three different growth methods were tried. The best results, yielding crystals up to several tens of micrometers in size, were obtained by slow vapor diffusion of a suited antisolved in a PbSe nanocrystal colloidal solution.

The obtained mesocrystals show a wealth of shapes. Nontwinned crystals are octahedron or trigon shaped, all bounded by {1 1 1} faces. Single and multiple twinning along {1 1 1} is very common, yielding platelets (mackles), 5-fold stars, and icosahedrons. These morphologies and the {1 1 1} twinning point to a cubic close-packed mesocrystal structure. Growth of the {1 1 1} facets proceeds by step flow involving undulated mono height steps as well as faceted macrosteps. Step sources are twins via the TPRE mechanism, 2D nucleation near the edges of the {1 1 1} faces, and contact nucleation induced by small crystallites on top of the facets. No evidence of spiral growth was found. Aside from twins, slip lines, and cracks, an interesting postgrowth phase transformation to body-centered cubic structures was often found, resulting in a zigzag groove pattern on the {1 1 1} crystal faces.

Our study shows that the nonclassical PbSe mesocrystal growth and its morphological consequences can well be described by the classical models of crystal growth, involving lateral growth via monoheight and macrosteps, different forms of two-dimensional nucleation, and effects resulting from impurity blocking. This despite the fact that the growth units are not atoms or molecules but are units 1 order of magnitude larger. The absence of spiral growth is the notable exception, which is a consequence of the absence or rarity of screw dislocations in the mesocrystal.

## Supplementary Material



## Notes

Materials used, synthesis of the quantum dots, and number of experimental details (PDF)
