# Supplementary material for: Growth and Morphology of PbSe Mesocrystals
Source: Cryst Growth Des. 2025 May 21;25(11):3894–905. doi: 10.1021/acs.cgd.5c00329 (PMC12142578; doi:10.1021/acs.cgd.5c00329)
Supplement: Supplementary file 1 [file cg5c00329_si_001.pdf]

Growth and morphology of PbSe mesocrystals

Paolo Accordini, Joeri Takke, Willem J.P. van Enckevort and Elias Vlieg*

Radboud University, Institute for Molecules and Materials,

Heyendaalseweg 135, 6525 AJ, Nijmegen, The Netherlands

e-mail: e.vlieg@science.ru.nl

SI 1 Materials

Lead acetate trihydrate (99.999%, Merck), Selenium (99.999%, Alfa Aesar), Oleic acid (OA, 90% Merck), Octadecene (ODE, 90% Merck), Diphenylphosphine (DPP, 98%, Merck), Trioctylphosphine (TOP, 97%, Merck), Acetone, Hexane (95% anhydrous, Merck), Toluene (99.8% anhydrous, Merck), Acetonitrile (99.8%, Merck), Chloroform ($>$ 99% anhydrous, Merck), Diethyl ether ($>=$ 99%, anhydrous), Tert-butyl methyl ether (99.8% anhydrous, Merck), Di-isopropyl ether (98%, Merck), Tetrahydrofuran ($>=$ 99.9% anhydrous, Merck), Ethyl acetate (99.8% anhydrous, Merck), Pyridine (99.8% anhydrous, Merck), Dimethylformamide (99.8% anhydrous, Merck), Nitrobenzene ($>=$ 99.9%), Methanol (99.8% anhydrous, Merck), Ethanol (anhydrous, Merck), 1-butanol (99.8% anhydrous, Merck), 1-propanol (99.7% anhydrous, Merck), 2-propanol (99.5% anhydrous, Merck). ODE was thoroughly degassed under vacuum over night before use. All other chemicals were used without any additional purification steps.

SI 2 Synthesis of PbSe quantum dots

PbSe nanocrystals used in this study were prepared using an adapted method derived from the synthesis route described by Steckel et al.[1]. Synthesis was performed in a Schlenk line using standard oxygen free techniques. For the lead oleate precursor: $11.38\,\mathrm{g}$ of lead acetate trihydrate (99.999% Aldrich) was mixed with $25.46\,\mathrm{g}$ oleic acid (OA, 90% Aldrich) and $37.91\,\mathrm{g}$ octadecene (ODE, 90% Aldrich) in a three-necked round bottom flask. This vessel was connected to the Schlenk line, its atmosphere was cycled with nitrogen and vacuum three times and heated up to $80\,^{\circ}\mathrm{C}$ under nitrogen atmosphere. Vacuum was applied when bubbling subsided and it was left degassing overnight to remove any trace of acetic acid. The trioctylphosphine selenide (TOPSe) precursor was prepared in a water and oxygen free glovebox (<1 ppm Oxygen and 1<ppm H20) by dissolving $2.52\,\mathrm{g}$ selenium (Se, 99.999% Alfa Aesar) in $16\,\mathrm{mL}$ of trioctylphosphine (TOP, 97% Aldrich). PbSe quantum dots were synthesized as follows: in the oxygen-free glovebox, a three necked flask was loaded with a stirring bar, $4.64\,\mathrm{g}$ of the lead precursor and $560\,\mathrm{mg}$ OA. ODE was added until reaching $10\,\mathrm{mL}$ total volume. A sealable syringe was prepared with a mixture of $8\,\mathrm{mL}$ TOPSe, $139\,\mu\mathrm{L}$ diphenyl phosphine (DPP, 98% Aldrich) and $2\,\mathrm{mL}$ ODE. The three necked flask was connected to the Schlenk line and its atmosphere was cycled three times. Then, the solution was heated up to $180\,^{\circ}\mathrm{C}$ and the selenium precursors was then swiftly injected in the flask. Temperature was kept constant at $130\,^{\circ}\mathrm{C}$ for $90\,\mathrm{s}$, after which $20\,\mathrm{mL}$ butanol was injected to quench the reaction. The vessel was allowed to cool down to room temperature before proceeding. Finally, $10\,\mathrm{mL}$ of methanol was added to force the precipitation of the quantum dots and the mixture was centrifuged at 2500 RPM for

10 minutes. Supernatant was discarded and the solid pellet was redispersed in fresh anhydrous toluene. This washing procedure was repeated 3 times before storing the nanocrystals in toluene/hexane solution in the glovebox for long term usage. The size distribution was estimated to be peaked around the radius of $4.3\,\mathrm{nm}$, estimated using direct TEM imaging and infrared spectroscopy[2].

SI 3 Damage by plastic deformation

Upon applying a gentle pressure, the PbSe mesocrystals deform easily as shown in the figure SI 1. This is a consequence of the weak van der Waals interactions between the aliphatic oleic tails of the ligands embedding adjacent PbSe quantum dots. So, the crystals have to be treated with care.

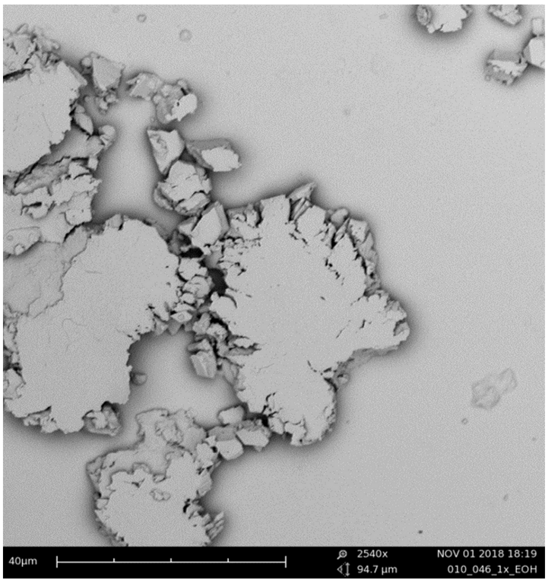

Figure SI 1: SEM micrograph of PbSe mesocrystals spread out by application of stress.

SI 4 X-ray diffraction pattern of a single PbSe mesocrystal

A PbSe single mesocrystal grown by the antisolvent evaporation method was selected and an X-ray diffraction pattern was recorded as shown in figure SI 2. The set-up employed was a Bruker D8 Quest diffractometer using Cu$K\alpha_1$ radiation. The vertical bright line in the middle of the image is an artifact introduced by the mounting of the two image halves. Diffraction rings of the PbSe crystals can be clearly recognized, pointing to considerable orientation disorder of the nanocrystallites in the mesocrystal. However, the rings are not uniform in intensity and some bright streaks can be seen. This indicates that some preferred crystallite orientation still exists. The indexing of the rings is based on a NaCl type lattice with axis length a $= 6.134\,\text{Å}$[3].

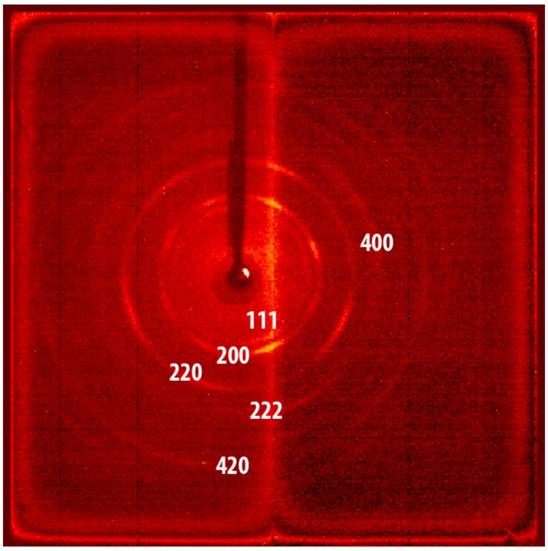

Figure SI 2: X-ray diffraction pattern of a PbSe single mesocrystal.

SI 5 Definition of size

Octahedrons as well as trigons were measured. For the octahedrons, size is determined by the edge length while for the trigons size is the length of the outer edge of the triangular crystals. This is indicated in the figures below.

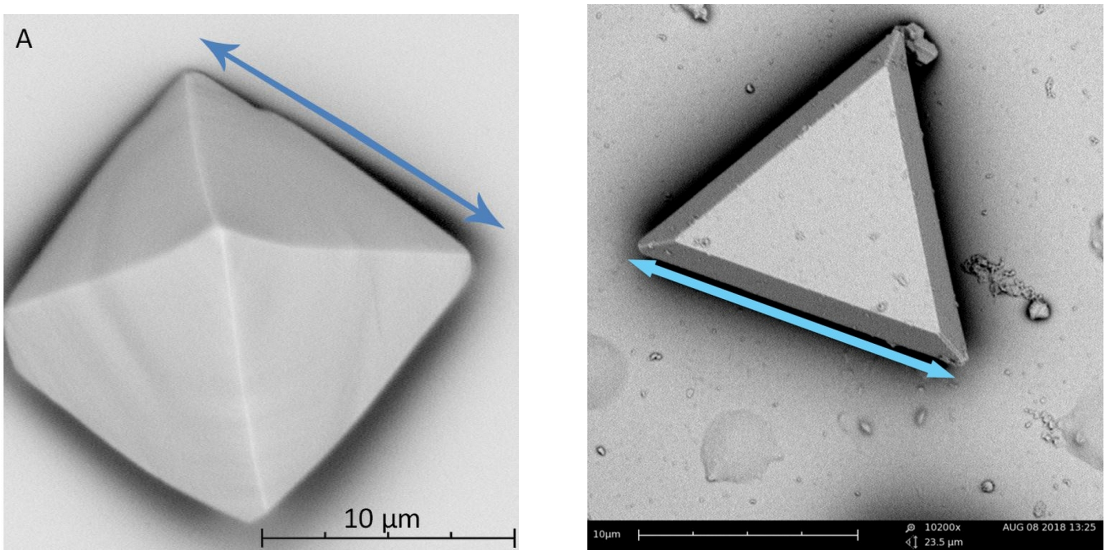

Figure SI 3: Definition of size of octahedral (**left**) and trigonal PbSe mesocrystals (**right**).

SI 6 AFM lattice imaging of PbSe mesocrystals after drop casting

The individual PbSe nanocrystals could be directly imaged using AFM, if deposited on a mica substrate after drop casting. This is shown in figure SI 4. The layer is obtained by evaporation of a $1\,\mu$L drop of a 1.5×10^{-5} molar nanocrystal solution in toluene. This was done in the glove box (< 1ppm H_2O and O_2) at room temperature. The size of the nanoparticles, including the ligands, is $8.1\,\mathrm{nm}$. This observation shows that it is feasible to image quantum dots directly by AFM, this in contrast to the mesocrystals of which the surface is obscured by the shut off effect[4]. The picture is recorded by AFM in phase sensitive mode employing a Dimension 3100 AFM (Digital instruments) using NSG-10 tips (TipsNano) with a radius of $10\,\mathrm{nm}$.

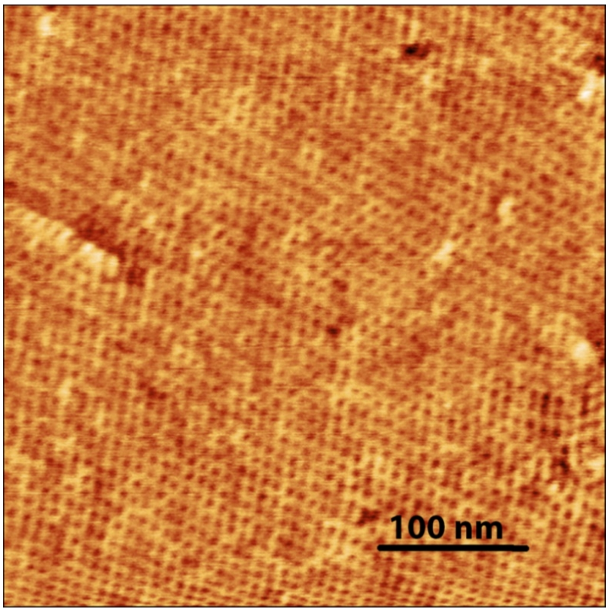

Figure SI 4: AFM image of PbSe nanocrystal layer obtained by drop casting on a mica substrate.

SI 7 Crystal with central hole in its surface

In a few cases, if the supersaturation was high, a central hole develops at the centre of some of the larger PbSe mesocrystallites. This is the result of an enhanced diffusion transport of growth units towards the outer parts of the crystal surface, which leads to a higher supersaturation and thus faster growth as compared to the middle part.

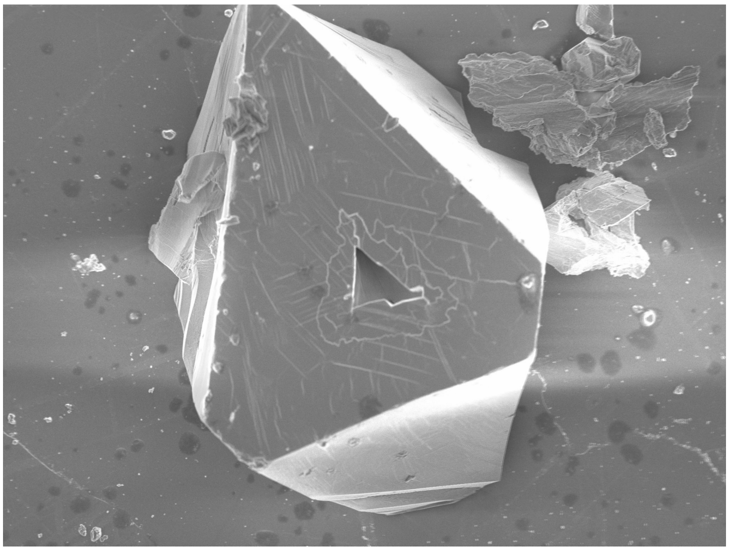

Figure SI 5: PbSe crystal with central hole in the (111) surface.

References

[1] J. S. Steckel, B. K. H. Yen, D. C. Oertel, and M. G. Bawendi. On the mechanism of lead chalcogenide nanocrystal formation. *JACS*, 128(40):13032–13033, 2006. ISSN 0002-7863. doi: 10.1021/ja062626g. URL https://doi.org/10.1021/ja062626g.

[2] I. Moreels, K. Lambert, D. De Muynck, F. Vanhaecke, D. Poelman, J. C. Martins, G. Allan, and Z. Hens. Composition and size-dependent extinction coefficient of colloidal pbse quantum dots. *Chem. Mat.*, 19(25):6101–6106, 2007. ISSN 0897-4756. doi: 10.1021/cm071410q. URL https://doi.org/10.1021/cm071410q.

[3] J. W. Earley. Description and synthesis of the selenide minerals*. *Amer. Miner.*, 35(5-6):337–364, 06 1950. ISSN 0003-004X.

[4] F. J. van den Bruele, K. M. Marks, B. Harmsen, A. L. Alfring, H. Sprong, W. J. P. van Enckevort, and E. Vlieg. Surface degradation during separation of crystals from solution: Minimizing the shut-off effect. *Cryst. Growth Des.*, 12(5):2265–2271, 2012. ISSN 1528-7483. doi: 10.1021/cg201506y. URL https://doi.org/10.1021/cg201506y.
